# Efficacy and safety of AZD7594, an inhaled non-steroidal selective glucocorticoid receptor modulator, in patients with asthma: a phase 2a randomized, double blind, placebo-controlled crossover trial

**DOI:** 10.1186/s12931-019-1000-7

**Published:** 2019-02-18

**Authors:** Mary N. Brown, Rainard Fuhr, Jutta Beier, Hong-Lin Su, Yingxue Chen, Henrik Forsman, Ulrika Wählby Hamrén, Helen Jackson, Ajay Aggarwal

**Affiliations:** 1 Respiratory, Inflammation and Autoimmunity Translational Medicine Unit, Early Clinical Development, IMED Biotech Unit, AstraZeneca, 35 Gatehouse Drive, Waltham, MA 02451 USA; 2 PAREXEL, International GmbH House 18, Klinikum Westend, Spandauer Damm 130, 14050 Berlin, Germany; 3 insaf – Respiratory Research Institute GmbH, Villa Berg Biebricher Allee 34, D-65187 Wiesbaden, Germany; 4 Biometrics, Early Clinical Development, IMED Biotech Unit, AstraZeneca, 35 Gatehouse Drive, Waltham, MA 02451 USA; 5 Quantitative Clinical Pharmacology, Early Clinical Development, IMED Biotech Unit, AstraZeneca, 35 Gatehouse Drive, Waltham, MA 02451 USA; 6 Quantitative Clinical Pharmacology, Early Clinical Development, IMED Biotech Unit, AstraZeneca, SE-43183 Gothenburg, Mölndal Sweden; 7 Respiratory, Inflammation and Autoimmunity Translational Medicine Unit, Early Clinical Development, IMED Biotech Unit, AstraZeneca, SE-43183 Gothenburg, Mölndal Sweden; 8 Respiratory, Inflammation and Autoimmunity IMED Biotech Unit, AstraZeneca, SE-43183 Gothenburg, Mölndal Sweden

**Keywords:** Asthma, Inhaled corticosteroids, Selective glucocorticoid receptor modulator, Pharmacokinetics, Cortisol

## Abstract

**Background:**

Inhaled corticosteroids reduce inflammation in asthma but chronic use may cause adverse effects. AZD7594, an inhaled non-steroidal selective glucocorticoid receptor modulator, has the potential of an improved risk-benefit profile. We investigated the safety and efficacy of AZD7594 in asthma.

**Methods:**

This phase 2a multi-center, randomized, double-blind, placebo-controlled crossover study enrolled adults with asthma aged 18 to 75 years. Patients were treated with budesonide 200 μg twice daily for 2–3 3 weeks (run in part one). If controlled, as demonstrated by an asthma control questionnaire-5 score of < 1.5, patients entered a three-week run-in (part two) where they received a short acting bronchodilator alone. Thereafter, patients with a fractional exhaled nitric oxide (F_E_NO) ≥25 ppb and pre-dose FEV_1_ 40 to 90% predicted were randomized to one of nine treatment sequences. Each patient received placebo and two of three dose levels of AZD7594 (58, 250, 800 μg) once daily via inhalation, in 14-day treatment periods, separated by three-week washout periods. The primary endpoint was the change from baseline in morning trough FEV_1_ versus placebo on day 15. Secondary endpoints included measures of airway inflammation and asthma control.

**Results:**

Fifty-four patients were randomized and received at least 1 dose of treatment, 48 patients completed the study. Overall 52 patients received placebo, 34 received AZD7594 58 μg, 34 received AZD7594 250 μg, and 34 received AZD7594 800 μg. AZD7594 800 μg demonstrated a significant improvement in Day 15 morning trough FEV_1_versus placebo (LS means difference 0.148 L 95% CI 0.035–0.261, *p* = 0.011), with a dose-dependent response seen in the 250 μg (0.076 L -0·036–0·188, *p* = 0.183) and 58 μg (0·027 L -0·086–0·140, *p* = 0.683). All secondary endpoints showed statistically significant improvement at the 800 μg dose. All doses demonstrated a significant reduction in F_E_NO at day 15 *p* < 0.01. No statistically significant difference in plasma cortisol level was observed between AZD7594 and placebo at any dose. AZD7594 was considered safe and well tolerated.

**Conclusions:**

Two-week treatment with AZD7594 demonstrated a favorable risk-benefit profile in patients with mild to moderate asthma. Further clinical studies are needed to fully characterize AZD7594.

**Trial registration:**

ClinicalTrials.gov number NCT02479412.

**Electronic supplementary material:**

The online version of this article (10.1186/s12931-019-1000-7) contains supplementary material, which is available to authorized users.

## Background

Asthma is characterized by chronic inflammation and glucocorticoid receptor (GR) agonists as inhaled corticosteroids (ICS) are the preferred anti-inflammatory treatment at all asthma severity levels [1]. ICS alone and in combination with long acting beta adrenergic agonists (LABAs) have demonstrated improvement in symptoms and lung function, reduction in exacerbations, improved asthma control and quality of life [2–4]. As ICS are administered topically and have low systemic absorption, the major limitation for ICS therapy is the risk of local adverse effects including candidiasis and dysphonia which are inconvenient for the patient, but typically not serious in nature. ICS therapy has the potential to cause systemic adverse effects mediated by the hypothalamic-pituitary-adrenal (HPA) axis, such as a reduction of growth velocity in children [5, 6] and effects on bone mineral density [7] although this effect is less clear [8]. Systemic effects are less common and usually associated with chronic use of high doses [9, 10].

Several development programs have aimed to improve selectivity for the glucocorticoid receptor to preserve efficacy while reducing adverse effects. Four compounds prior to AZD7594 have advanced to clinical trials in asthma [11], but none have advanced beyond phase 2 trials.

AZD7594 is a novel nonsteroidal, potent and selective glucocorticoid receptor modulator (SGRM) being developed as a once daily inhaled treatment for asthma. Pre-clinical data using the Rat Sephadex®-induced lung edema model of pulmonary inflammation supports an improved therapeutic ratio when delivered by inhalation [12]. In this phase 2a randomized study, the first to evaluate clinical efficacy, we aimed to assess the efficacy, safety, and pharmacokinetics (PK) of AZD7594 given once daily by inhalation in adults with asthma uncontrolled on short acting beta agonists (SABAs) alone. We investigated the effect of AZD7594 on morning trough FEV_1_, airway inflammation, and measures of asthma control. Some results of this study were presented at the European Respiratory Society meeting in 2017, [13, 14] this paper provides the complete results from the study.

## Methods

### Study design

**Fig. 1:**
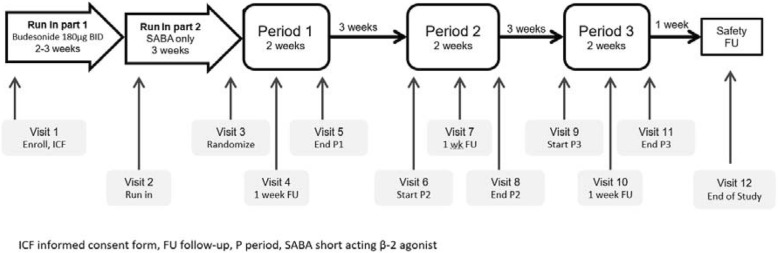
Trial design

This phase 2 randomized, double-blind, multi-dose, placebo controlled, three-period, incomplete block crossover study was conducted in nine centers in Germany and one center in Bulgaria between June 25, 2015 and February 8, 2016 (Fig. 1).

### Patients

Eligible patients were men and women of non-child bearing potential 18 to 75 years of age with mild to moderate asthma. Patients must have had a documented clinical diagnosis of asthma at least 6 months prior to enrollment and were either steroid naïve or treated with monteleukast, ICS or low dose ICS / LABA. Current or recent smokers (who quit within the last 6 months) and patients with ≥10 years total smoking history were excluded. Patients were required to have a fraction of exhaled nitric oxide (F_E_NO) level ≥ 25 parts per billion (ppb) before randomization, demonstrating evidence of airway inflammation. Full inclusion and exclusion criteria are provided in the Additional file 1: appendix.

### Randomization and masking

Eligible patients were randomized via an interactive voice response system prior to the first dose in treatment period one, to one of nine treatment sequences according to the randomization schedule produced by independent PAREXEL Informatics. Each treatment sequence included two out of three possible treatments: AZD7594 58 μg, AZD7594 250 μg, AZD7594 800 μg, and each patient received placebo during one of the three periods in a balanced crossover design. The study was performed in a double-blind manner. Placebo was supplied as identical dry powder vehicle to AZD7594 in identical packaging, and budesonide (used during run-in) and salbutamol (rescue medication) were open label.

### Treatment

During a two to 3 week run-in (part one) patients received low dose budesonide (200 μg twice daily). Patients whose asthma was controlled (as demonstrated by an Asthma Control Questionnaire-5 [ACQ-5] score ≤ 1.5), were eligible for a second run-in (part two) during which budesonide was stopped and washed out over 3 weeks while patients received only the SABA, salbutamol, as needed. At the end of the second run-in, patients with pre-bronchodilator forced expiratory volume in 1 second (FEV_1_) between 40 to 90% and with a F_E_NO of ≥25 ppb were randomized and entered the double-blind treatment period. Patients who were poorly controlled as evidenced by rescue medication use of ≥12 puffs on three or more days or an ACQ-5 ≥ 3 during the enrollment period were excluded. Randomized patients received study medication once daily by inhalation via a monodose dry powder inhaler (DPI) for a 2 week treatment period. Each treatment period was separated by a three-week washout, where the only asthma treatment that patients received was as needed salbutamol. A stability criterion was applied at baseline of treatment periods 2 and 3 to ensure that there was no detectable carry-over effect or any acute worsening compared to baseline at the start of those periods. Patients were evaluated to confirm that the pre-dose FEV_1_ was ±20% of the pre-dose baseline value for treatment period 1.

### Endpoints

The primary efficacy endpoint was change from baseline in morning trough FEV_1_ on Day 15 versus placebo for each of the three AZD7594 dose levels. Baseline was defined as the average of the measurements obtained at 60 and 30 min before dosing on day 1 in each treatment period, and trough as the average of the 2 spirometric measurements at 23:00 and 23:30 h after the last dose of study medication on Day 14. Spirometry was assessed centrally.

Secondary efficacy endpoints included change from baseline in: F_E_NO on Day 8 and Day 15, morning trough FEV_1_ on Day 8, and in morning and evening peak expiratory flow (mPEF, ePEF) over the treatment period. Patient reported outcomes for asthma control were assessed with the five item asthma control questionnaire (ACQ-5) recorded at each study visit. Asthma symptoms, night-time awakenings, rescue medication use and lung function as measured by morning and evening peak flow were recorded daily in the eDiary. Asthma symptoms were captured twice daily using a four point scale where patients recorded 0 for no symptoms and 3 if their asthma prevented them from doing normal daily activities or sleeping. The eDiary data was used to calculate the number of symptom free days, defined as those days on which no asthma symptoms or night-time awakenings occurred and the number of asthma control days which included the same criteria as symptom free days and no rescue medication use. The pharmacokinetic (PK) profile was also assessed as a secondary endpoint, with rich sampling in a subset of patients, blood samples were collected on Day 1 and Day 14 of each treatment period. PK parameters, C_max_ and AUC_0–24_ were derived using non-compartmental methods with Phoenix® WinNonlin® (Pharsight Corp., Mountain View, California, USA). In addition, the dose proportionality was assessed by dose normalized C_max_ and AUC_0–24_ across three dose levels. A 24 h plasma cortisol profile was performed on Day − 1 and Day 14, in the PK subset, for each study period to obtain AUEC_0–24_ pre-treatment and post-treatment respectively, to evaluate cortisol suppression.

### Statistical analysis

Thirty-six patients were needed to achieve approximately 88% power to detect 200 mL change from baseline in trough FEV_1_ compared to placebo using a two-sided test at 5% significance level, assuming an intra-patient standard deviation (SD) of 210 mL. Assuming a dropout rate of 25%, 48 patients randomized to nine sequences were needed to ensure 36 evaluable patients completing the study.

The analysis of the efficacy endpoints was based on the full analysis set (FAS), defined as all randomized patients following the principle of intention to treat (ITT) that includes all randomized patients who received at least one dose of randomized study drug. Patients were included in the analysis according to the treatment to which they were randomized. All safety analyses were based on the safety analysis set (SAF) defined as all randomized patients who received at least one dose of randomized study drug during the Treatment Period. Classified by actual treatment received.

The efficacy endpoints were analyzed using a linear model with treatment, period, and sequence as fixed effects, baseline as continuous covariate, and patient within sequence as random effect. No multiplicity adjustment for the analysis of the secondary variables was performed. In addition, dose-response for trough FEV_1_ day 15 was analyzed *posthoc* using nonlinear mixed-effects modeling. In this analysis an empirical Emax model was estimated to the change from baseline trough FEV_1_ data, applying different baseline definitions (see Additional file 1). This study is registered with ClinicalTrials.gov, number NCT02479412.

## Results

One hundred ten patients were enrolled and screened, 54 of whom were randomized. Thirty-four patients received AZD7594 58 μg, 34 received AZD7594 250 μg, 34 AZD7594 800 μg and 52 patients received placebo. Baseline demographics characteristics of participants were similar across treatment groups. (Table 1). Trough PK was collected in all study participants, 15 were included in an intensive PK sampling subset. Forty-eight patients (89%) completed the study, four withdrew due to development of pre-specified study withdrawal criteria. and One patient was withdrawn due to randomization into the wrong PK group and one for an adverse event (vitamin B12 deficiency and elevated liver enzymes). Three patients had major protocol deviations and were excluded from the per protocol set (failed inclusion criteria, use of prohibited concomitant medication, and withdrawn without primary endpoint). Patients recorded daily in the eDiary if they took their study medication. Drug accountability records were kept by the investigator and remaining supplies, including spent capsules were sent to the sponsor. Across all treatment groups, mean compliance was > 96%.

**Table 1:** Baseline demographic and clinical characteristics

	Placebo	AZD7594		
	(*n* = 52)	58 μg (*n* = 34)	250 μg (*n* = 34)	800 μg (*n* = 34)
Demographics				
Age, years Mean (SD)	51 (12)	51 (12)	50 (12)	51 (12)
Male *n* (%)	44 (84.6%)	26 (76.5%)	30 (88.2%)	28 (82.3%)
White *n* (%)	51 (98.1%)	33 (97.1%)	33 (97.1%)	34 (100%)
Black *n* (%)	1 (1.92%)	1 (2.94%)	1 (2.94%)	0 (0.00)
BMII kg/m^2^ Mean (SD)	27 (3)	27 (4)	28 (3)	27 (3)
Asthma Characteristics				
Prebronchodilator FEV_1_ (L), Mean (SD)	2.53 (0.74)	2.54 (0.73)	2.59 (0.72)	2.55 (0.69)
% patients meeting FEV_1_ reversibility criteria^a^	78.8%	88.2%	73.5%	70.6%
ACQ-5 score mean (SD)	1.2 (0.7)	1.2 (0.6)	1.2 (0.6)	1.2 (0.7)
Asthma symptom score (SD)	0.8 (0.5)	08 (0.5)	0.8 (0.5)	0.8 (0.5)
F_E_NO ppb (SD)	57 (45)	60 (47)	53 (34)	57 (39)

^a^Reversibility was defined as ≥12% and ≥ 200 ml increase from pre-bronchodilator FEV1, *ACQ-5* Asthma Control Questionnaire-5, *BMI* body mass index, *F*_*E*_*NO* Fraction exhaled nitric oxide, *kg* kilogram, *m* meter, *SD* standard deviation

### Efficacy

**Fig. 2:**
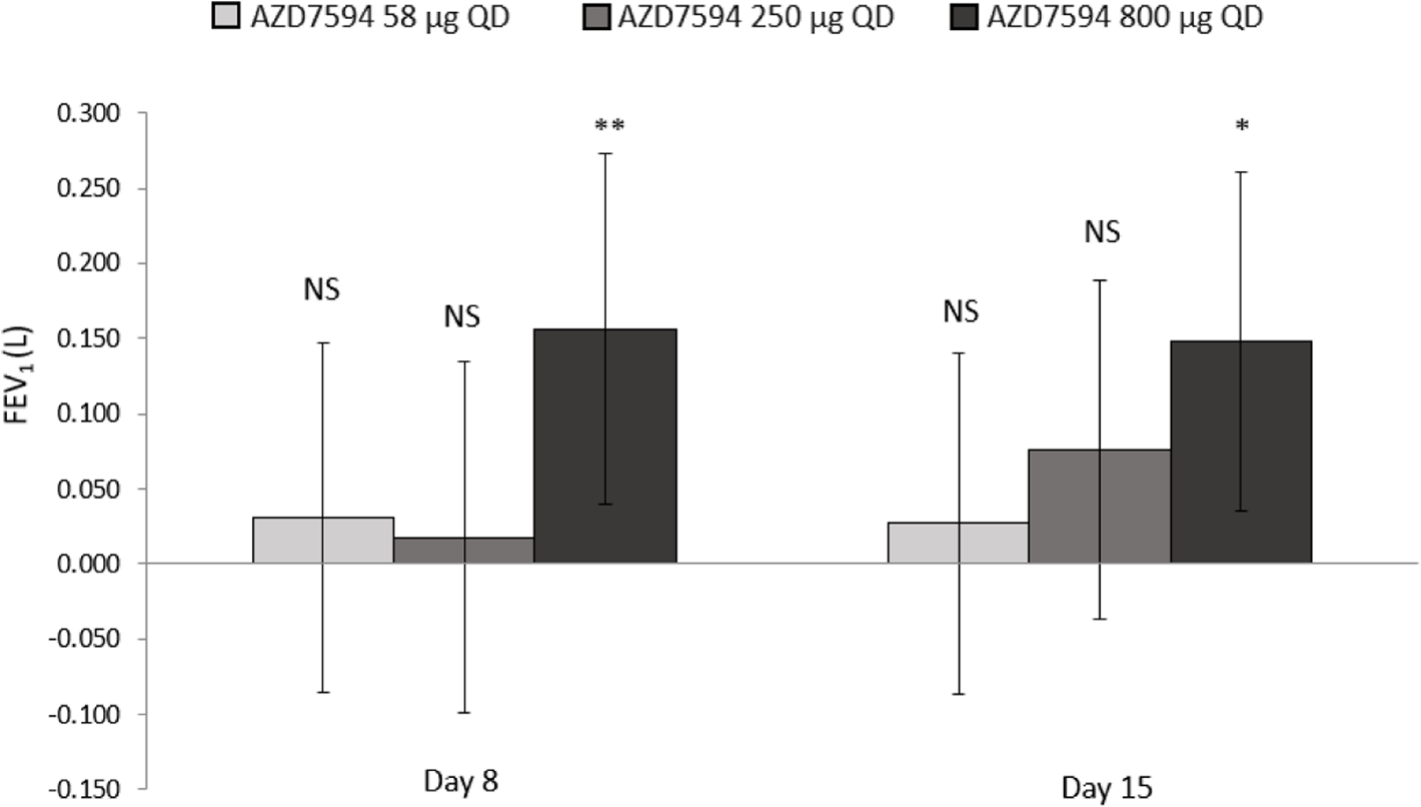
Least squares mean change from baseline versus placebo in trough FEV_1_ L (full analysis set). Baseline was defined separately for each treatment period as the average of the 60 and 30 minutes pre-dose measurements taken on day 1 of each treatment period. Error bars represent 95% confidence intervals. * *p* = 0.011, ** *p* = 0.009, *QD* once daily, *FEV*_1_ forced expiratory volume in 1 second, *L* liters, *NS* not significant

Lung function improved at all doses (Fig. 2). The greatest improvement in the primary endpoint, the change from baseline in morning trough FEV_1_ on Day 15 versus placebo, occurred with the 800 μg dose (LS Means difference 0.148 L 95% CI 0.0349 to 0.261 *p* = 0.011) (Table 2).

**Table 2:** Efficacy endpoints Least Squares Means full analysis set

	Placebo	AZD7594 58μg	AZD7594 250μg	AZD7594 800μg
	(*n* = 52)	(*n* = 34)	(*n* = 34)	(*n* = 34)
Primary efficacy endpoint				
Morning pre‐bronchodilator FEV_1_				
Change from baseline (L) on day 15	0.059	0.086	0.136	0.207
Treatment difference (L) (95% CI)	N.A.	0.027 (‐0.086, 0.140)	0.076 (‐0.036, 0.188)	0.148 (0.035, 0.261)
P value	N.A.	0.638	0.183	0.011
Key secondary efficacy endpoints				
Morning pre‐bronchodilator FEV_1_				
Change from baseline (L) on day 8	0.071	0.102	0.089	0.227
Treatment difference (L) (95% CI)	N.A.	0.031 (‐0.086, 0.147)	0.017 (‐0.010, 0.134)	0.156 (0.039, 0.273)
P value	N.A.	0.604	0.767	0.009
Key secondary efficacy endpoints				
Morning pre‐bronchodilator FEV_1_				
Change from baseline (L) on day 8	0.071	0.102	0.089	0.227
Treatment difference (L) (95% CI)	N.A.	0.031 (‐0.086, 0.147)	0.017 (‐0.010, 0.134)	0.156 (0.039, 0.273)
P value	N.A.	0∙604	0∙767	0∙009
Fraction exhaled nitric oxide (F_E_NO)				
Change from baseline (ppb) on day 8	‐4.296	‐9.153	‐14.71	‐19.04
Treatment difference (ppb) (95% CI)	N.A.	‐4.857 (‐11.24, 1.528)	‐10.41 (‐16.75, ‐4.075)	‐14.75 (‐21.18, ‐8.319)
P value	N.A.	0.134	0.002	<0.0001
Change from baseline (ppb) on day 15	‐0.549	‐14.40	‐14.81	‐20.44
Treatment difference (ppb) (95% CI)	N.A.	‐13.85 (‐24.06, ‐3.642)	‐14.26 (‐24.37, ‐4.149)	‐19.90 (‐30.10, ‐9.689)
P value	N.A.	0.008	0.006	0.0002
Morning peak expiratory flow (mPEF)				
Change from baseline Day 1‐14	0.0814	10.42	5.334	12.60
Treatment difference (L/min) (95% CI)	N.A.	10.34 (‐1.335, 22.01)	5.253 (‐6.427, 16.93)	12.52 (0.748, 24.29)
P value	N.A.	0.082	0.374	0.037
Evening peak expiratory flow (ePEF)				
Change from baseline Day 1‐14	‐8.257	7.475	6.04	11.65
Treatment difference (L/min) (95% CI)	N.A.	15.73 (5.039, 26.43)	14.30 (3.534, 25.06)	19.91 (9.068, 30.75)
P value	N.A.	0.004	0.010	0.0004
Forced vital capacity (FVC)				
Change from baseline (L) on day 8	0.0844	0.062	0.084	0.153
Treatment difference (L) (95% CI)	N.A.	‐0.023 (‐0.137, 0.091)	‐0.0003 (‐0.115, 0.114)	0.068 (‐0.046, 0.183)
P value	N.A.	0.694	0.996	0.240
Change from baseline (L) on day 15	0.0765	0.042	0.104	0.138
Treatment difference (L) (95% CI)	N.A.	‐0.035 (‐0.141, 0.072)	0.028 (‐0.078, 0.134)	0.062 (‐0.045, 0.168)
P value	N.A.	0.521	0.598	0.254
Asthma symptom score				
Change from baseline day 1 ‐ 14	‐0.012	‐0.119	‐0.094	0.215
Treatment difference (95% CI)	N.A.	‐0.107 (‐0.206, ‐0.008)	‐0.082 (‐0.182, 0.018)	‐0.203(‐0.303, ‐0.103)
P value	N.A.	0.035	0.105	0.0001
Asthma symptom free days				
Change from baseline day 1 ‐ 14	0.050	0.603	0.318	1.01
Treatment difference (95% CI)	N.A.	0.553 (0.036, 1.069)	0.268 (‐0.252, 0.788)	0.956 (0.433, 1.48)
P value	N.A.	0.036	0.308	0.0005
Asthma control days				
Change from baseline day 1 ‐ 14	0.277	0.950	0.705	1.219
Treatment difference (95% CI)	N.A.	0.673 (0.088, 1.258)	0.428 (‐0.161, 1.017)	0.942 (0.348, 1.535)
P value	N.A.	0.025	0.152	0.002
ACQ‐5 day 15				
Change from baseline	0.014	‐0.293	‐0.168	‐0.416
Treatment difference (95% CI)	N.A.	‐0.307 (‐0.516, ‐0.098)	‐0.182 (‐0.393, 0.028)	‐0.430 (‐0.640, ‐0.221)
P value	N.A.	0.004	0.088	<0.0001
Rescue medication use (day 1 – 14)				
Change from baseline (inhalations) per day	‐0.334	‐0.678	‐0.819	‐1.137
Treatment difference (95% CI)	N.A.	‐0.344 (‐0.719, 0.0318)	‐0.485 (‐0.863, ‐0.1073)	‐0.803 (‐1.183, ‐0.422)
P value	N.A.	0.072	0.012	<0.0001
Nighttime awakenings				
Change from baseline day 1‐ 14	0.007	‐0.412	‐0.173	‐0.760
Treatment difference (95% CI)	N.A.	‐0.419 (‐0.741, ‐0.956)	‐0.179 (‐0.503, 0.144)	‐0.766 (‐1.091, ‐0.441)
P value	N.A.	0.0116	0.273	<0.0001

ACQ‐5 Asthma Control Questionnaire‐5, CI confidence interval, FEV_1_ forced expiratory volume in 1 second, L liters, ppb parts per billion

**Fig. 3:**
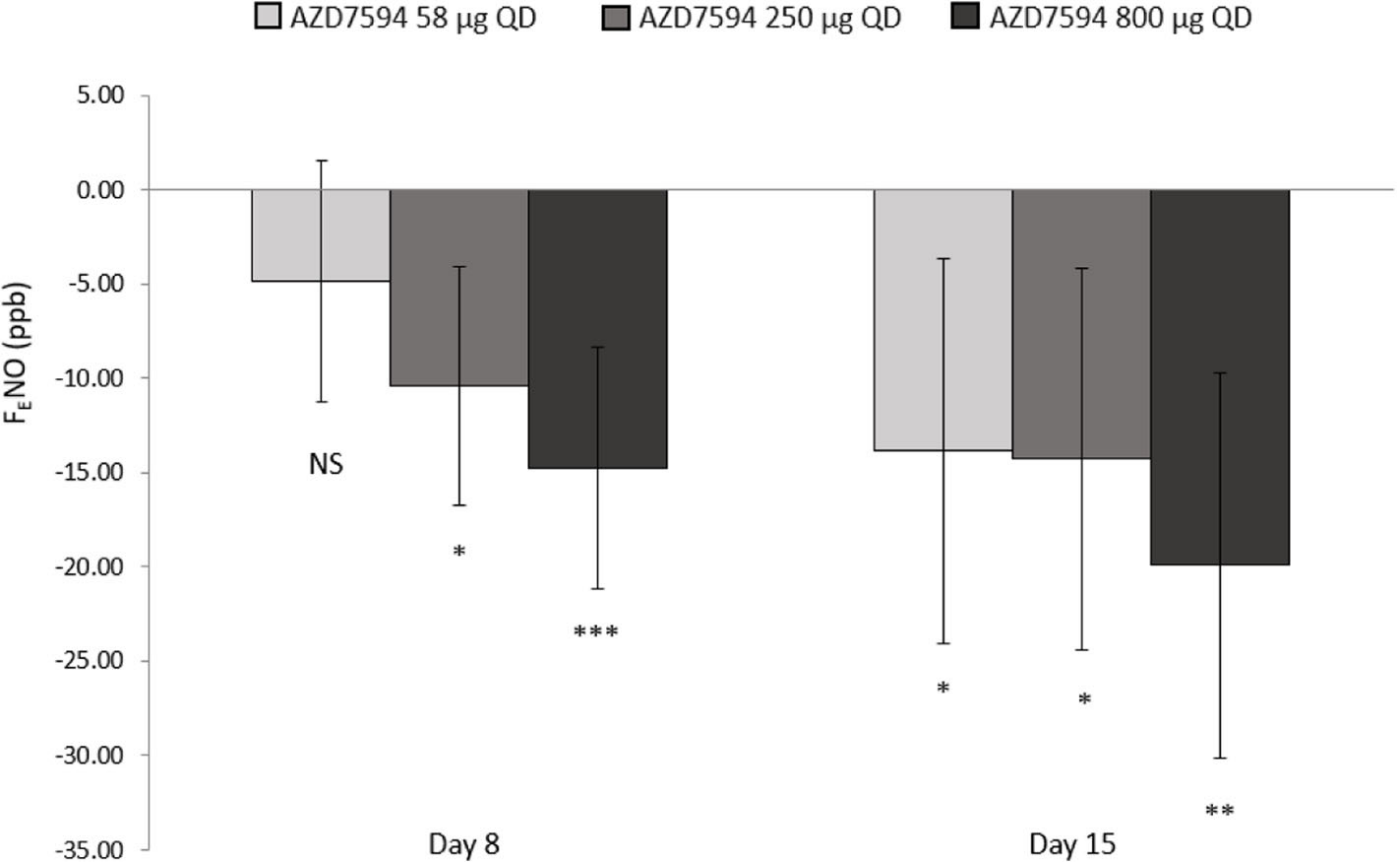
F_E_NO Least square mean change from baseline versus placebo (full analysis set). Baseline was defined separately for each treatment period as the measurement performed on day 1 of each treatment period. Error bars represent 95% confidence intervals. * *p* <0.01, ** *p* = <0.001, *** *p* < 0.0001 *QD* once daily, *F*_*E*_*NO* fraction exhaled nitric oxide, *ppb* parts per billion, *NS* not significant

**Fig. 4:**
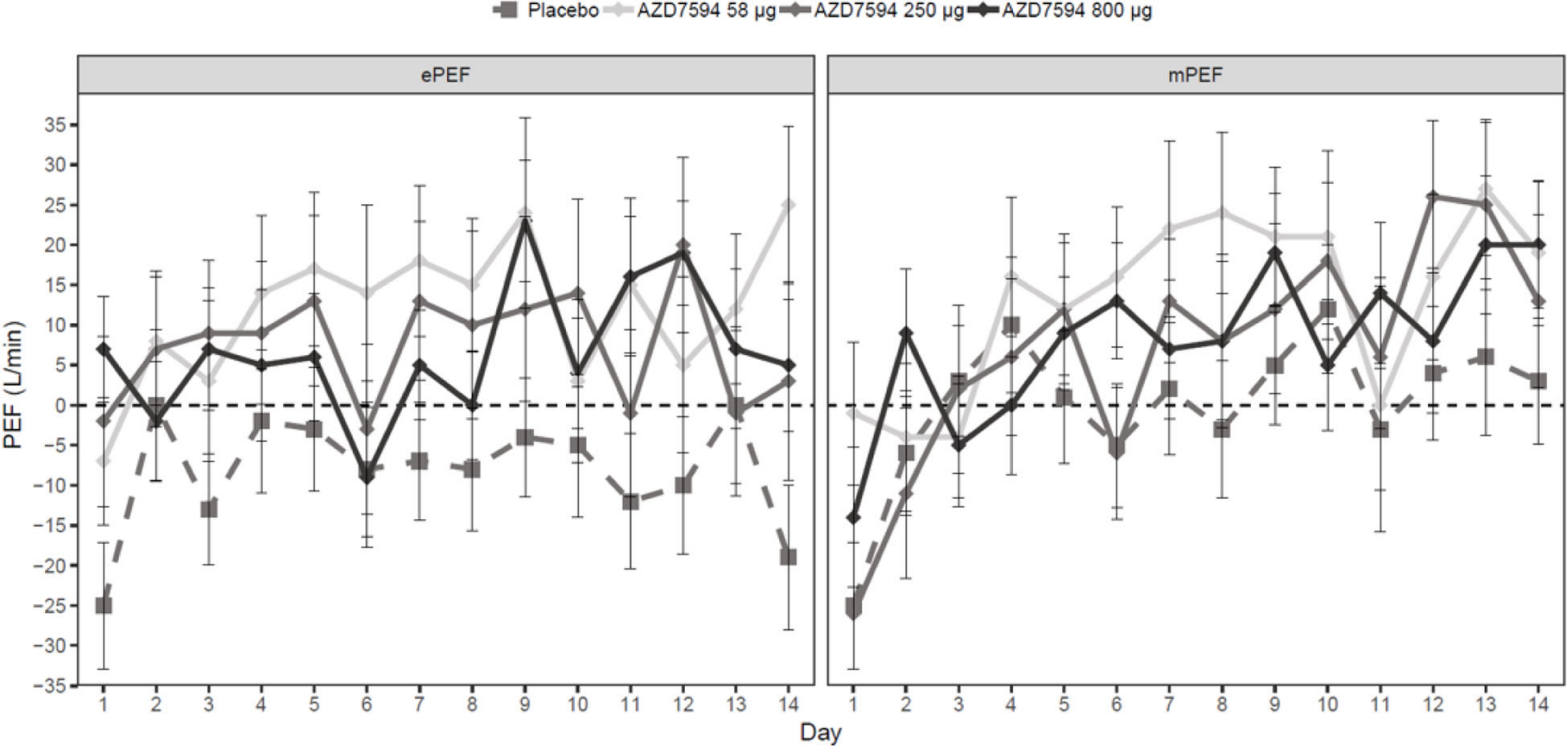
ePEF (left) and mPEF (right) Arithmetic mean change from baseline versus day (full analysis set). Baseline is defined as the average of the Day -1 and Day -7 during the run-in period. Error bars represent standard errors. ePEF evening peak expiratory flow, mPEF morning peak expiratory flow

**Fig. 5:**
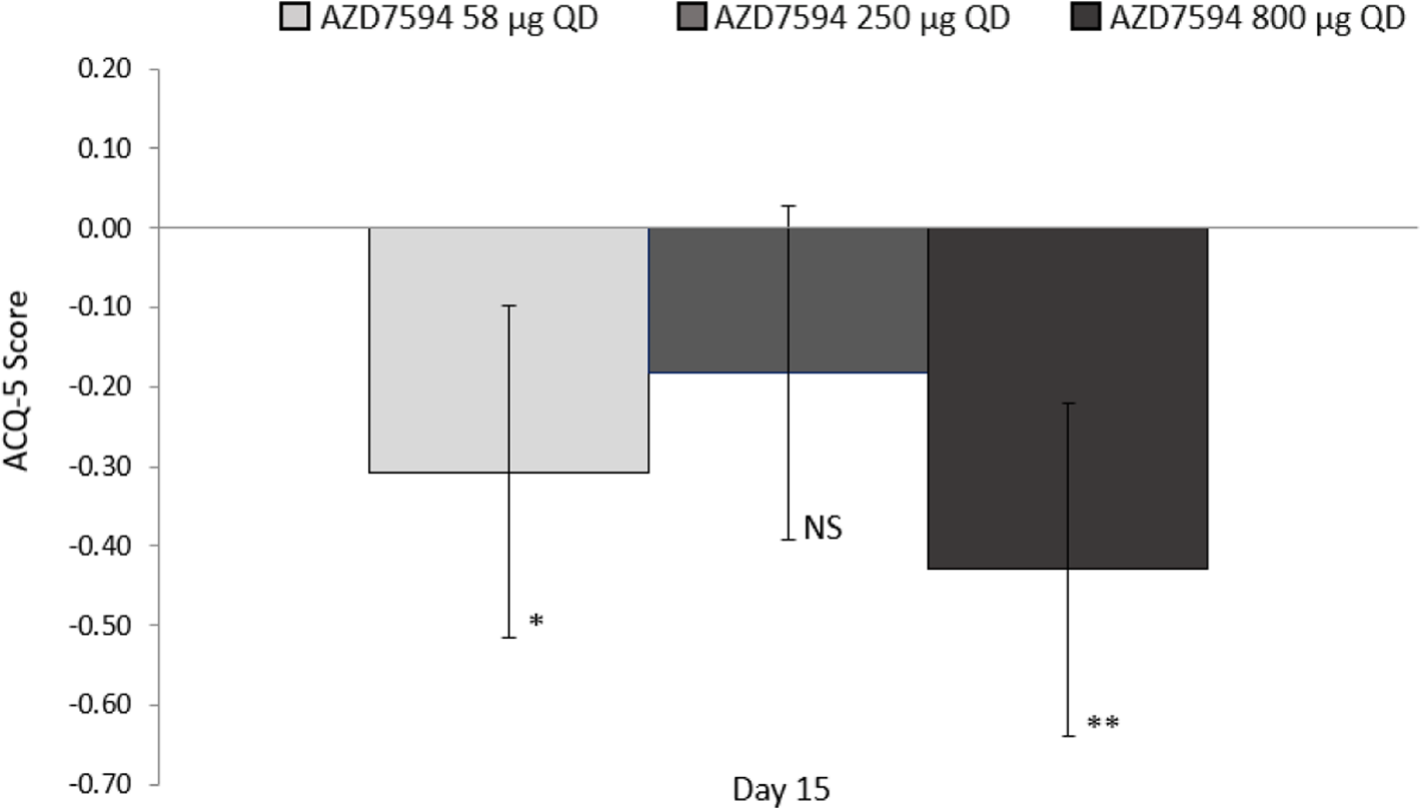
ACQ-5 Least squares mean change from baseline versus placebo on Day 15 (full analysis set). Baseline was defined separately for each treatment period as the pre-dose score on day 1 of each treatment period. Error bars represent 95% confidence intervals. * *p* < 0.05, ** *p* < 0.001, *NS* not significant, *QD* once daily, *ACQ-5* Asthma Control Questionnaire-5

The 800 μg dose also showed the greatest improvement in change from baseline in morning trough FEV_1_ versus placebo on Day 8 (LSMeans difference 0.156 L 95% CI 0.039 to 0.273 *p* < 0.01). The change from baseline in F_E_NO values demonstrated a dose ordered improvement (Fig. 3), with the 250 μg and 800 μg doses significant versus placebo at Day 8 *p* < 0.05 and a significant treatment difference across all doses at Day 15 p < 0.01(Table 2). Over the course of the treatment period, evening PEF improved relative to placebo at all dose levels (*p* < 0.01) while morning peak flow was significantly improved only with the AZD7594 800 μg dose level (Fig. 4, Table 2). The greatest reduction in average (Day 1 to Day 14) daily use of rescue medication from baseline occurred at the 800 μg dose (LS Means difference − 0.80 *p* < 0.0001) and the 250 μg dose also demonstrated a significant reduction (− 0.49 *p* = 0.012). Asthma control improved, as evidenced by significant improvement in mean ACQ-5 score versus placebo at Day 15 in both the 58 μg and 800 μg doses, (Fig. 5). Both symptom free days and asthma control days as well as reduction in nighttime awakenings were significant versus placebo with AZD7594 800 μg (Table 2).

In the *posthoc* analysis, the dose-response in change from baseline trough FEV_1_ was described by an empirical Emax model (Additional file 1). An adequate fit could only be achieved when the average baseline across all periods was used. The ED_50_ was estimated to 140 [95%CI 26–763] μg, and the Emax to 0.213 [0.078–0.348] L. The model predicted trough FEV_1_ Day 15 versus placebo is shown in Additional file 1: Figure S1. The predicted effects are numerically larger, compared to the primary analysis.

### Pharmacokinetics

**Fig. 6:**
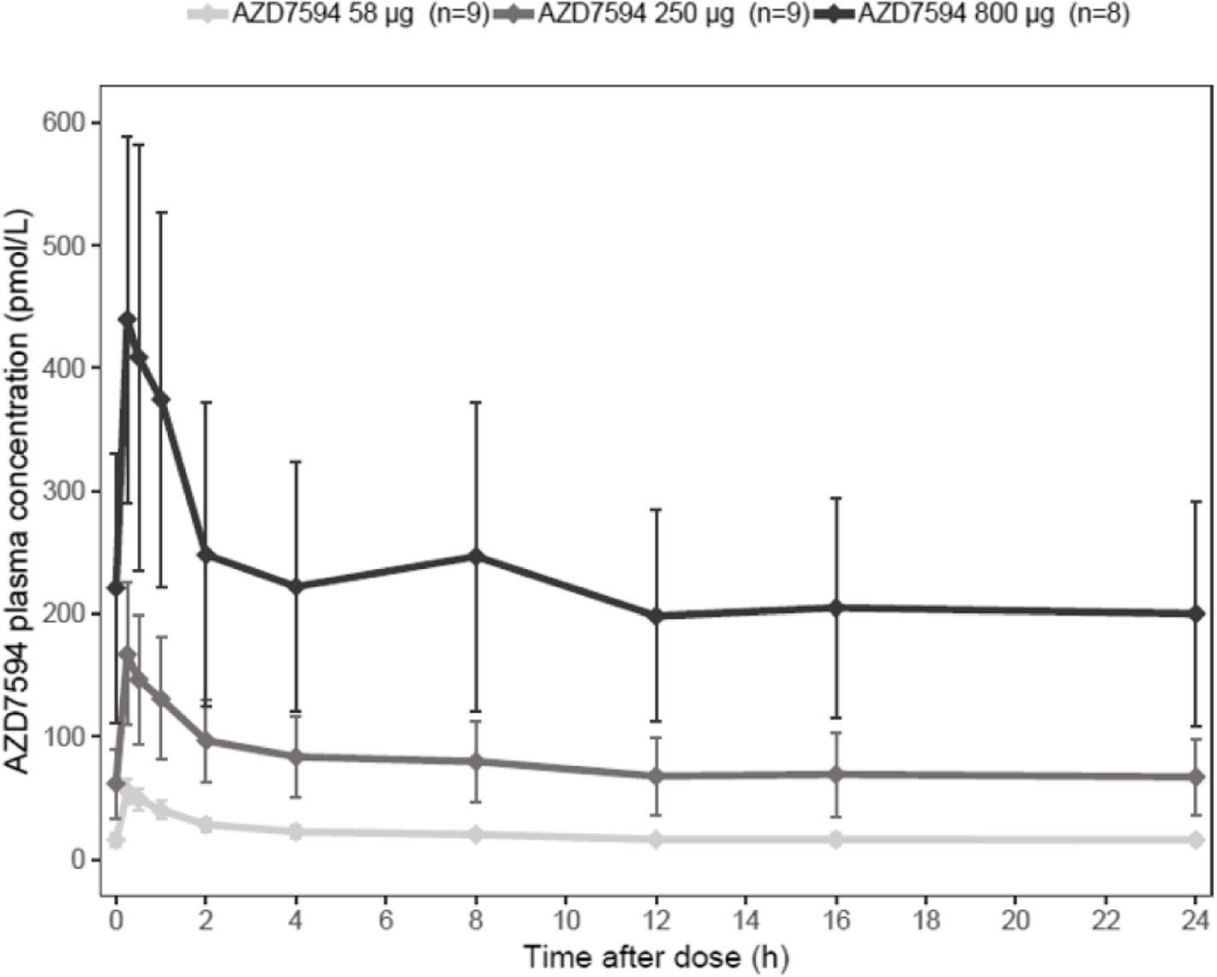
AZD7594 Plasma concentrations at Steady-state (Day 14) Arithmetic means by dose level vs time after dose (pharmacokinetic analysis set). Error bars represent standard deviation. n: number of subjects

Following inhalation administration, AZD7594 was rapidly absorbed and reached maximum plasma concentration within 15 min; this was followed by a rapid decline in plasma concentration over 2 h, and then a slow elimination phase. The observed AZD7594 concentration-over-time profiles for all three dose levels are shown in Fig. 6. The increases in AZD7594 mean systemic exposures [C_max_ and AUC_0–24_ values] were less than dose proportional as indicated by the dose-normalized values (C_max_/D, C_max,ss_/D and AUC_(0–24)/_D). There was moderate to high between-subject variability in AZD7594 PK parameters (Table 3). The geometric coefficient of variation (CV%) ranged from 19.70 to 43.21% in C_max_ (Days 1 and 14), and from 17.91 to 52.48% in AUC_0–24_.

**Table 3:** Summary of the PK Parameters of AZD7594 at Steady‐State (Day 14) by Dose Levels

Geometric mean	AZD7594	AZD7594	AZD7594
(CV%)	58 μg	250 μg	800 μg
	(*n* = 9)	(*n* = 9)	(*n* = 8)
Cmax, ss (pmol/L)	54.97 (19.70)	158.7 (35.01)	421.6 (37.26%)
AUC(0‐24) (h×pmol/L)	467.1 (17.91)	1725 (44.33)	4894 (52.48%)
Cavg, ss (pmol/L)	19.48 (17.93)	71.89 (44.33)	203.9 (52.55%)

AUC(0‐24): area under the plasma concentration‐time curve from time zero to 24 hours after dose administration; Cavg,ss: average plasma concentration during a dosing interval at steady‐state; Cmax,ss: observed maximum plasma concentration at steady‐state; CV%: geometric coefficient of variation; n: number of patients in the PKS for each treatment

### Safety and tolerability

AZD7594 was well tolerated. Adverse events (AEs) were reported by 17 (32%) of patients in the placebo group, 13 (38%) of patients in the AZD7594 58 μg group, 9 (26%) in the 250 μg group and 12 (35%) in the 800 μg group (Table 4). There were no severe adverse events (SAEs) or deaths during the study. One patient was discontinued due to an AE of pernicious anemia (vitamin B12 deficiency anemia) and increased hepatic enzymes noted on the blood safety samples drawn at the end of the first washout period after receiving placebo in period one. No clinically important differences were seen between treatments with regard to the number and overall pattern of patients reporting adverse events by system organ class. Adverse events are summarized in Table 4. Nasopharyngitis and headache were the most commonly reported AEs. No statistically significant difference in plasma cortisol level versus placebo was observed for any dose level of AZD7594, geometric mean ratio 1·03 (95% CI 0.90–1.19) for 58 μg, 1.02 (0.88–1.18) for 250 μg and 0.91 (0.79–1.05) for 800 μg (Table 5).

**Table 4:** Treatment emergent adverse events reported by ≥2 patients in any dose level (safety analysis set)

Adverse Event	Placebo	AZD7594 58 μg	AZD7594 250 μg	AZD7594 800 μg
	*N* = 52	*N* = 34	*N* = 34	*N* = 34
	n (%)	n (%)	n (%)	n (%)
Patients with any AE	17 (32.69)	13 (38.24)	9 (26.47)	12 (35.29)
Nasopharyngitis	8 (15.38)	4 (11.76)	2 (5.88)	4 (11.76)
Headache	0 (0.00)	0 (0.00)	3 (8.82)	2 (5.88)
Gastroenteritis	0 (0.00)	3 (8.82)	0 (0.00)	0 (0.00)
Diarrhoea	0 (0.00)	0 (0.00)	0 (0.00)	3 (8.82)
Cough	1 (1.92)	2 (5.88)	0 (0.00)	0 (0.00)
Dyspnoea	2 (3.85)	0 (0.00)	0 (0.00)	0 (0.00)

MedRA version 18.1, AE adverse event

**Table 5:** AUEC0‐24 for plasma cortisol on Day 14

Dose	n	Geometric mean ratio to baseline compared to placebo (95% CI)	*p*‐value
placebo	14		
58 μg	9	1.03 (0.90‐1.19)	0.6342
250 μg	9	1.02 (0.88‐1.18)	0.7620
800 μg	9	0.91 (0.79‐1.05)	0.2008

AUEC 0‐24 Area under plasma concentration time curve from time zero to 24 hours after dose administration, CI confidence Interval

## Discussion

This phase 2a study was the first to evaluate clinical efficacy of AZD7594 administered once daily via inhalation for 2 weeks in patients with mild to moderate asthma. AZD7594 reduced lung inflammation and improved lung function as evidenced by a clinically and statistically significant improvement in the primary endpoint, Day 15 trough FEV_1_ versus placebo at the 800 μg dose, improved daily peak flow and asthma symptoms, reduced nocturnal awakenings and average daily rescue medication use.

Treatment with AZD7594 was associated with a significantly lower inflammatory burden as measured by F_E_NO compared to placebo. F_E_NO levels dropped in a dose-ordered manner at Day 8 and after 2 weeks of treatment all AZD7594 doses achieved a significant difference versus placebo *p* < 0.01. A clinically meaningful mean reduction of > 20% [15] was observed for all dose levels, with the greatest F_E_NO reduction (35%) occurring at the 800 μg dose. As an effective biomarker of eosinophilic airway inflammation with a greater predictive value than spirometry and PEF in assessing ICS responsiveness in asthma, the clinical improvement observed, provides confidence in the anti-inflammatory properties of AZD7594 across a dose range [15, 16]. Though this study measured many common asthma endpoints as well as F_E_NO as marker of inflammation, other endpoints of interest for future studies include markers of inflammation in sputum, as well as direct measurement of bronchial hyperresponsiveness.

The objective of the study was to assess efficacy and tolerability across a wide dose range. Although the two lower doses did not significantly improve trough FEV_1_, there was a numerical improvement at all dose levels. A *posthoc* analysis conducted to gain a better understanding of dose-response showed a dose-dependent increase in trough FEV_1_, although the uncertainty in dose-response is large as can be expected for a GR agonist [17, 18]. Lung function improved significantly at all doses as assessed by ePEF and at the 800 μg dose for mPEF. Peak flow has the advantage over spirometry of being assessed daily. In this study, improvements in peak flow measurements were accompanied by a reduction in symptoms and nighttime awakenings and an increase in symptom free days, all important indicators of asthma control. The correlation of peak flow and asthma control has been previously reported [19].

In this study, ACQ-5 scores improved significantly with both the low (58 μg) and high (800 μg) dose of AZD7594. Although improvement was shown, a conclusion regarding the clinically meaningful effect with this relatively small sample over a 2 week treatment cannot be drawn.

The current crossover study in a small number of subjects, was designed to assess the potential of AZD7594 as an anti-inflammatory treatment rather than fully characterize the clinical profile. A two part run in ensured appropriate patient selection for treatment with a GR agonist alone. By including patients who had an ACQ-5 of ≤1.5 on low dose ICS as assessed during run in part one, we aimed to include patients at GINA step 2,3 [1]. Inclusion of patients who had a minimum F_E_NO of 25 ppb at the end of run-in part two enabled the enrollment of patients who demonstrated airway inflammation and were more likely to respond to a GR agonist; American Thoracic Society (ATS) guidelines suggest patients with F_E_NO levels lower than 25 ppb are unlikely to respond to GR agonist therapy [15]. The selection of a responder patient population provided more confidence in evaluating anti-inflammatory effect in a small number of subjects.

The 14-day treatment period allows adequate time to achieve clinical response. Previous studies demonstrate within 2 weeks near maximal improvement in lung function, as measured by FEV_1,_ [2, 20, 21] and reduction of F_E_NO, a measure of airway inflammation. [15, 22, 23]. Lung function may have continued to improve with a longer treatment period. Personal best peak flow is typically reached within 1 to 3 weeks of initiating GR agonist therapy, but daily average peak flow may continue to improve for 3 months or more [24]. In future studies, a longer treatment period will be preferred to assess asthma control.

The three-week washout was supported by the pharmacokinetic profile of AZD7594 [25], and glucocorticoid receptor agonist studies which demonstrate both FEV_1_ and F_E_NO return to baseline levels within 14 to 21 days [20–22]. To limit the potential of any detectable carry-over effect, a baseline stability criterion was included for each period. The results should be interpreted with care since no correction for multiple testing was done for the secondary variables. For many of the secondary endpoints, the 250 μg dose resulted in a lower response than the lowest dose. This is likely a result of variability and a small sample size, and dose-response modeling provides further support to select doses to investigate in the future studies.

Overall, in this study, AZD7594 was safe and well tolerated. The incidence of AEs was similar across treatment groups, the most common AE was nasopharyngitis. While cortisol suppression has been seen at higher doses of AZD7594 [26], only limited cortisol suppression was observed at the highest dose in this study, no significant difference versus placebo was observed. The absence of candidiasis and dysphonia in all patients is encouraging but a longer treatment period is needed to more fully assess the adverse effect profile.

Although the current study met its objective of assessing short term efficacy and safety of AZD7594, a parallel design study over a longer treatment period in broader patient population is needed to adequately assess the minimally efficacious and optimal doses. A novel once daily inhaled non-steroidal glucocorticoid modulator with an improved therapeutic risk-benefit profile would be a welcome addition to current asthma therapies, further clinical studies, including comparison to an active ICS comparator, are needed to fully characterize AZD7594.

## Conclusions

In this phase 2a study, the first to evaluate clinical efficacy in patients with mild to moderate asthma, once daily inhaled administration of AZD7594, a novel non-steroidal selective GR modulator demonstrates improved lung function as measured by FEV_1_ and PEF, a reduction of airway inflammation as evidenced by a reduction in F_E_NO and a positive impact on symptoms and asthma control. AZD7594 was well tolerated with an adverse effect profile similar to placebo. This study supports that a once daily inhaled non-steroidal SGRM is effective and well tolerated and may provide an alternative to ICS for patients in the future. Further clinical work is required to fully characterize the clinical profile and compare to currently available ICS.

## Additional file

Additional file 1: Supplementary appendix. (PDF 175 kb)

## Abbreviations

ACQ-5: Asthma Control Questionnaire-5
AE: Adverse Effect
AMP: Adenosine monophosphate
ATS: American Thoracic Society
AUC(0-24): Area under the plasma concentration-time curve from time zero to 24 hours after dose administration
AUEC(0-24): Area under the effect curve from time zero to 24 hours after dose administration
BID: Twice daily
CI: Confidence interval
Cmax: Observed maximum plasma concentration
Cmax, ss: Observed maximum plasma concentration at steady-state
Cmax, ss/D: Dose-normalized observed maximum plasma concentration at steadystate
Cmax/D: Dose-normalized observed maximum plasma concentration
CV%: Geometric coefficient of variation
DPI: Dry powder inhaler
[ED]50: Dose that produces the desired therapeutic effect in 50% of the patients
Emax: Maximum effect
ePEF: Evening peak expiratory flow
FAS: Full Analysis Set
F_E_NO: Fractional exhaled nitric oxide
FEV_1_: Forced expiratory volume in 1 second
GINA: Global Initiative for Asthma
GR: Glucocorticoid receptor
HPA: Hypothalamic-pituitary-adrenal
ICS: Inhaled corticosteroids
IEC: Independent Ethics Committee
IRB: Institutional Review Board
ITT: Intention to treat
L: Liter
LABA: Long-acting β-2 agonist
mPEF: Morning peak expiratory flow
PEF: Peak expiratory flow
PK: Pharmacokinetic
ppb: Parts per billion
SABA: Short-acting β-2 agonist
SAE: Serious Adverse Effect
SAF: Safety Analysis Set
SD: Standard deviation
SGRM: Selective glucocorticoid receptor modulator
