# Supplementary material for: Efficacy and safety of AZD7594, an inhaled non-steroidal selective glucocorticoid receptor modulator, in patients with asthma: a phase 2a randomized, double blind, placebo-controlled crossover trial
Source: Respir Res. 2019 Feb 18;20:37. doi: 10.1186/s12931-019-1000-7 (PMC6380015; doi:10.1186/s12931-019-1000-7)

Supplementary appendix

Efficacy and safety of AZD7594, an inhaled non-steroidal selective glucocorticoid receptor modulator, in patients with asthma: A Phase 2a randomized, double blind, placebo controlled crossover trial.

Authors: Mary N. Brown, Rainard Fuhr, Jutta Beier, Hong-Lin Su, Yingxue Chen, Henrik Forsman, Ulrika Wählby Hamrén, Helen Jackson, Ajay Aggarwal

Table S1 Inclusion Criteria

Patients will be entered into this study only if they meet all of the following criteria; however, each of Visits 1 to 3 can be repeated once within 1 week of the original visit, but only if the patient does not meet selected inclusion criteria (i.e., FEV1, reversibility, FeNO or ACQ-5), and the Investigator believes that a repeat visit may allow the patient to become eligible to enter the study:

1. Provision of informed consent before any study specific procedures
2. Body mass index of 18 to 35 kg/m2
3. Men and women 18 to 75 years of age, inclusive
4. Patients need to be nonsmokers or ex-smokers (quit ≥ 6 months before Visit 1) with total smoking history of less than 10 pack years.
5. Documented clinical diagnosis of asthma for ≥ 6 months before Visit 1
6. Patients on low dose ICS (equivalent of budesonide ≤ 400 µg per day) or low-dose ICS/LABA, or not on any inhaled steroids, or patients on montelukast
7. Patients should be controlled on low dose budesonide during the first 2-3 weeks of Run-in Part 1, i.e., they need to have ACQ-5 of ≤ 1.5 at Visit 2.
8. Prebronchodilator FEV1 at Visit 3 should be between 40% and 85% of predicted (mean of 2 predose measurements taken 30 minutes apart).
9. All patients need to have reversibility to salbutamol (per American Thoracic Society/European Respiratory Society [ATS/ERS] criteria, 2005) on Visit 1. Patients who are on ICS/LABA at the time of enrollment (Visit 1), need to have ICS/LABA stopped before reversibility testing. They can be started on budesonide Run-in, and Visit 1 reversibility testing can be done after approximately 48 hours of last LABA use. A repeat Visit 1 may be done within 1 week of the original visit; patients must demonstrate reversibility (≥ 12% and ≥ 200 mL change in FEV1.
10. All patients need to have FeNO concentrations of ≥ 25 parts per billion (ppb) at Visit 3.
11. Demonstrate the ability to use the study inhalation device properly
12. For optional inclusion in the genetic component of the study, patients must provide informed consent for the genetic sampling and analysis
13. Women must be of nonchildbearing potential defined as meeting 1 of the following criteria:
    - Permanently or surgically sterilized, including hysterectomy and/or bilateral oophorectomy and/or bilateral salpingectomy
    - Postmenopausal; aged ≤ 50 years and have been amenorrheic for 12 months or more following cessation of exogenous hormonal treatments and with luteinizing hormone (LH) and follicle stimulating hormone (FSH) levels in the postmenopausal range
    - Postmenopausal; aged ≥ 50 years and have been amenorrheic for 12 months or more, following cessation of all exogenous hormonal treatments
14. Male patients should be willing to use a condom to prevent pregnancy and exposure of a female partner to AZD7594 and should refrain from donating sperm or fathering a child from the first day of dosing until 3 months after the last dose of IMP.

Table S2 Exclusion Criteria

Patients will be entered into this study only if they meet none of the following criteria:

1. Known or suspected hypersensitivity to the IMPs or excipients, including lactose
2. Systemic steroid use in the 6 weeks before Visit 1
3. Any active disease other than asthma
4. Patients on medium to high dose ICS (equivalent of budesonide > 400µg per day) or on inhaled anticholinergic combination within the 6 weeks prior to Visit 1
5. Compliance with the eDiary of at least 80% of the days is expected in both Run-in and Treatment Periods. Patients with < 80% eDiary compliance during Run-in Periods would not be randomized
6. Treatment with biologicals such as monoclonal antibodies or chimeric biomolecules including omalizumab within 6 months or 5 half-lives before Visit 1, whichever is longer
7. History or clinical suspicion of any clinically relevant disease or disorder which, in the opinion of the Investigator, may either put the patient at risk because of participation in the study, or influence the results or the patient's ability to participate in the study, or any other safety concerns in the opinion of the Investigator
8. ACQ-5 ≥ 3 at any time between Visits 1 and 3
9. Any clinically important abnormalities in rhythm, conduction or morphology of the dECG at rest and any abnormalities in the 12-lead dECG that, as considered by the Investigator, may interfere with the interpretation of QTc interval changes.
10. Prolonged QT interval using Fridericia's correction (QTcF) ≥ 450 msec or family history of long QT syndrome.
11. PR (PQ) interval prolongation (> 240 msec), intermittent second or third degree atrial-ventricular (AV) block or AV dissociation
12. Patients with implantable cardiac defibrillator and patients with sustained symptomatic ventricular and/or atrial tachyarrhythmia
13. Patients with unstable angina pectoris or stable angina pectoris classified higher than Canadian Cardiovascular Society Class II, or a myocardial infarction or stroke within 6 months before Visit
14. Any contraindication against the use of vagolytic or sympathomimetic drugs as judged by the Investigator
15. History of hospitalization within 12 months caused by heart failure or a diagnosis of heart failure higher than New York Heart Association Class II
16. Patients with hepatitis B surface antigen (HBsAg), hepatitis C virus (HCV) antibody or human immunodeficiency virus (HIV)
17. Donation of blood (≥ 450 mL) within 3 months or donation of plasma within 14 days before Visit 1
18. Suspected poor capability to follow instructions of the study, as judged by the Investigator
19. Previous participation or prior screen failure in the current study, or participation in any other research study in the 1 month before Visit 1
20. History of or current alcohol or drug abuse (including marijuana), as judged by the Investigator
21. Planned in-patient surgery, major dental procedure or hospitalization during the study
22. Pregnant woman or a nursing mother
23. Involvement in the planning and/or conduct of the study (applies to both AstraZeneca staff, contract research organization staff and/or staff at the study site)
24. Suspicion of Gilbert's syndrome
25. Vulnerable persons (e.g., persons kept in detention)
26. ACQ-5 of ≥ 3 or daily rescue use of ≥ 12 puffs for ≥ 3 consecutive days during the enrollment period

Posthoc dose-response analysis of trough FEV_1 day 15

To describe the dose-response in change from baseline trough FEV_1 on day 15, an empirical Emax model was estimated using NONMEM version 7.3 (ICON Development Solutions, Ellicott City, MD, USA). In this analysis, different baselines were considered. The baseline definition for the primary analysis of trough FEV_1 (pre-dose for each period) was first used. Secondly, to reduce variability in baseline, the average of the trough FEV_1 baselines across all treatment periods for each individual was employed. An empirical Emax model was used, estimating the effect at zero dose (E_0), the maximum effect (Emax) and the dose giving half-maximum effect (ED_{50}):

$$\text{Effect} = E_0 + \text{Emax}*\text{DOSE}/(ED_{50}+\text{DOSE})$$

An adequate model fit could only be achieved when the average baseline across periods was used. The ED_{50} was estimated to 140 [95%CI 26-763] µg, and the Emax to 0.213 [0.078-0.348] L. The linear model used for the primary analysis, but applying the average baseline across periods, was also estimated for comparison.

Figure S1 Emax model predicted mean (95%CI) difference from placebo in trough FEV_1 on Day 15 (blue) with estimated effects using a linear model with average baseline across the periods as a covariate (black points with error bars).

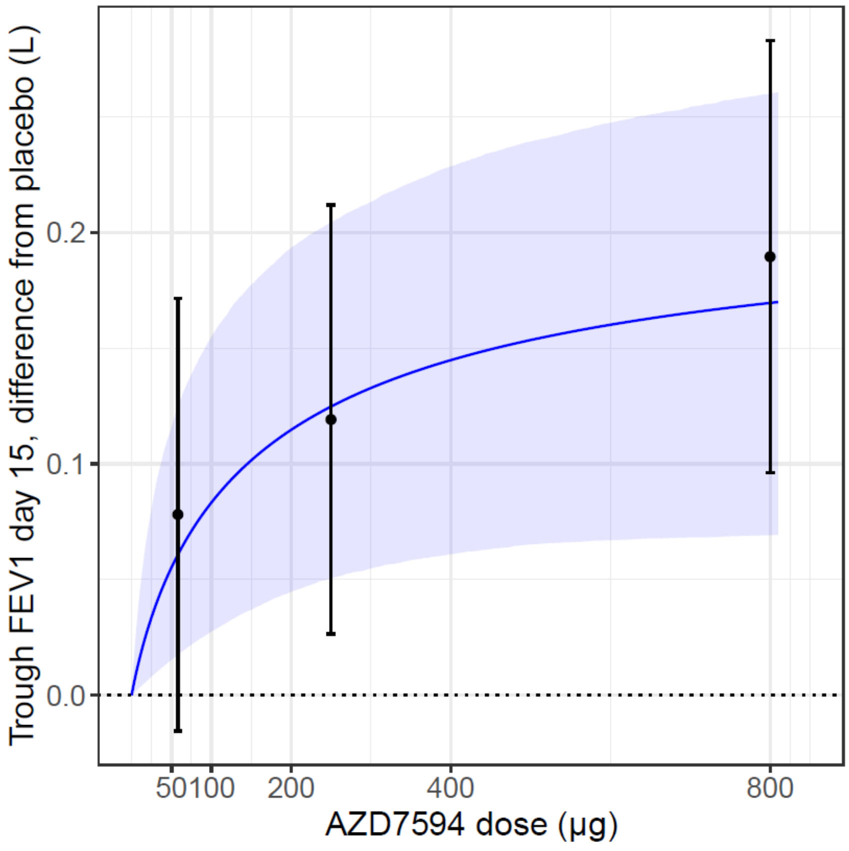

Supplement: Supplementary file 1 — Supplementary appendix. (PDF 175 kb) [file 12931_2019_1000_MOESM1_ESM.pdf]
